# Hydrogels as a Replacement Material for Damaged Articular Hyaline Cartilage

**DOI:** 10.3390/ma9060443

**Published:** 2016-06-03

**Authors:** Charlotte M. Beddoes, Michael R. Whitehouse, Wuge H. Briscoe, Bo Su

**Affiliations:** 1 School of Oral and Dental Sciences, University of Bristol, Lower Maudlin Street, Bristol BS1 2LY, UK; C.Beddoes@bristol.ac.uk; 2 Musculoskeletal Research Unit, University of Bristol, Level 1 Learning and Research Building, Bristol BS10 5NB, UK; michael.whitehouse@bristol.ac.uk; 3 School of Chemistry, University of Bristol, Cantock’s Close, Bristol BS8 1TS, UK; Wuge.Briscoe@bristol.ac.uk

**Keywords:** articular cartilage, hydrogels, self-healing, implant, double network

## Abstract

Hyaline cartilage is a strong durable material that lubricates joint movement. Due to its avascular structure, cartilage has a poor self-healing ability, thus, a challenge in joint recovery. When severely damaged, cartilage may need to be replaced. However, currently we are unable to replicate the hyaline cartilage, and as such, alternative materials with considerably different properties are used. This results in undesirable side effects, including inadequate lubrication, wear debris, wear of the opposing articular cartilage, and weakening of the surrounding tissue. With the number of surgeries for cartilage repair increasing, a need for materials that can better mimic cartilage, and support the surrounding material in its typical function, is becoming evident. Here, we present a brief overview of the structure and properties of the hyaline cartilage and the current methods for cartilage repair. We then highlight some of the alternative materials under development as potential methods of repair; this is followed by an overview of the development of tough hydrogels. In particular, double network (DN) hydrogels are a promising replacement material, with continually improving physical properties. These hydrogels are coming closer to replicating the strength and toughness of the hyaline cartilage, while offering excellent lubrication. We conclude by highlighting several different methods of integrating replacement materials with the native joint to ensure stability and optimal behaviour.

## 1. Hyaline Cartilage Structure

Hyaline cartilage is produced by chondrocytes. The cells secrete proteoglycan and collagen fibres, creating a microenvironment known as a chondron [1,2] that combine to form the cartilage matrix. In articular joints, the hyaline cartilage partially interpenetrates into the porous subchondral layer of the bone, coating its surface. The thickness of the hyaline cartilage, measured as the distance from cartilage surface to the underlying bone, varies by joint. As an example, cartilage at the ankle has an average thickness of ~1.2 mm, while at the knee, the average cartilage is almost twice as thick (~2.2 mm) [3]. Hyaline cartilage is highly deformable, to facilitate an increased contact area with the opposing surface and distribute the applied load more effectively with the surrounding cartilage [4]. The articular cartilage is repaired by collagen and proteoglycans that are secreted from the chondrocytes. However, due to the cartilage’s avascular nature, poor nutrient supply, and slow waste extraction that is reliant on diffusion through the cartilage matrix, the metabolic activity of the cells are reduced resulting in prolonged repair times.

**Figure 1:**
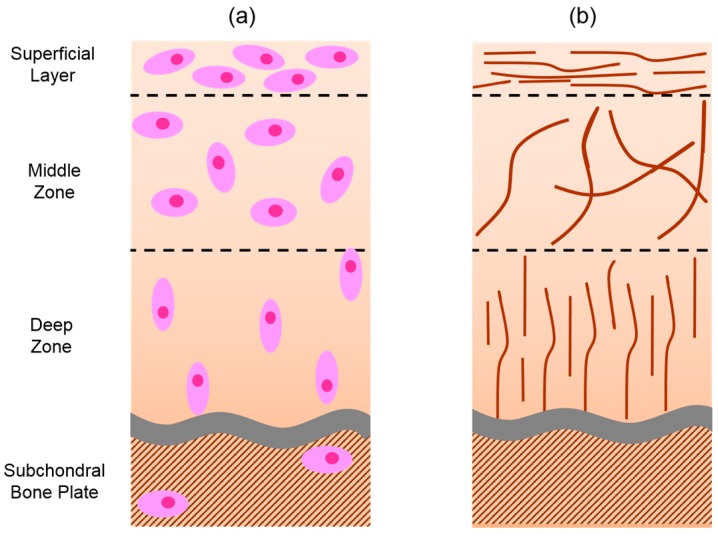
Orientation and structure of (**a**) chondrocytes; and (**b**) collagen fibres within the different layers of the articular hyaline cartilage.

The structure of cartilage has been extensively examined by a number of different techniques, including light microscopy, X-ray tomography [5], scanning electron microscopy [6], and cryo- scanning electron microscopy [7]. Compared to other tissue, the cell content of the articular cartilage is low [8], with chondrocytes making up only 1–5 vol % of the entire matrix [9]. The most abundant material in cartilage is water, which occupies 65–80 vol %, decreasing with increasing depth [10,11]. The large amount of water present in cartilage assists in the movement of nutrients and waste molecules, while reducing the friction of movement and supporting the cartilage’s deformation during an applied load. Collagen and proteoglycans are present in the articular cartilage in approximately equal volumes of 10–20 wet wt % each [9]. The collagen (primarily type II) provides the tensile strength, while also assisting in the attachment of the chondrocytes to the matrix. The proteoglycans provide the compressive strength and enable the accommodation of the large water content. This is achieved by their charged anionic sulphate and carboxylate groups, which attract cationic ions, and thus the water molecules, via an osmotic effect [12]. Articular cartilage is anisotropic and inhomogeneous, consisting of distinct layers: subchondral bone, deep zone, middle zone, calcified cartilage zone, and the superficial bone plate (Figure 1).

The superficial layer equates to 10%–20% of the cartilage’s total thickness, making it the thinnest of the four main layers [13]. The chondrocytes are deformed into oval shapes, which remain aligned parallel to the surface. This region is directly exposed to the tensile, compressive, and shear stresses, and so contains a high concentration of collagen fibres, which also align parallel to the surface. The dense layer of collagen has low fluid permeability, generating a large resistance to rapid water loss [14]. With the majority of the water molecules unable to depart from the cartilage, the built up fluid pressure is a contributing factor to the load support [15] and prevents compression of the chondrocytes [13]. In the case of bovine carpometacarpal cartilage, >90% of a 130 kPa load was supported by the water pressure [16]. The water retention ability varies between the different layers of hyaline cartilage, for example, the superficial layer of human knee cartilage was able to support up to ~74% of a peak average stress of 1.35 MPa, whereas the deep zone supported only 53% [17].

Below the superficial layer is the middle zone, which equates to 40%–60% of the cartilage’s total thickness [13]. The collagen concentration is lower, enabling randomly orientated thicker fibres. The proteoglycan concentration is at its highest within this layer (25% dry weight), resulting in greater swelling pressures and a higher water content. The chondrocyte concentration is lower, and they are more rounded in shape, with relatively higher synthetic activity when compared to the superficial layer [18]. The alignment of the cells are also reduced, with approximately two-thirds of the cells remaining horizontal, while a third align vertically [9].

The third layer is the deep zone that corresponds to ~30% of the total cartilage thickness. This layer contains the lowest concentration of cells, which are deformed elliptically and are aligned vertically. The most distinguishing feature of this layer is the collagen fibres, which are woven together, perpendicular to the surface.

The calcified cartilage zone acts as an interface between cartilage and subchondral bone. The calcified cartilage is mineralized, which increases the stiffness of the material when compared to the previous layers. This layer has several functions, including attaching the cartilage to the bone and reducing the diffusion of materials from the bone into the cartilage [19]. Rather than flat, this layer forms a tidemark-like structure [20,21], which accounts for ~3%–8% of the cartilage thickness [22]. This zone can be further divided into three layers: tidemark, calcified cartilage, and cement line. The tidemark is a mineralised front and allows a gradual transition to the calcified cartilage [23], while the cement line separates the calcified cartilage from the subchondral plate [23]. 

The final layer is the subchondral bone plate, the outer layer of the subchondral bone. This layer secures the cartilage to the joint’s surface by infusing itself into the porous structure of the bone. The cartilage remains a highly porous structure, increasing the bone density by only ~15% [24]. Nutrients are transported from the subchondral bone to the cartilage with the use of blood vessels that protrude into the calcified cartilage zone [23]. Below this plate is the subchondral trabecular bone structure and the remaining bone.

## 2. Causes of Cartilage Damage 

Articular cartilage can be damaged by high or rapidly applied loads, commonly from either a sporting injury [25] or an abrupt impact [26,27]. Alternatively, cartilage can also be damaged at a slower rate by interfacial and fatigue wear [13]. This type of wear is commonly developed from a considerable amount of repetitive motion that is more common in certain occupations such as: textile weavers [28], dancers [29], miners [30], and other manual workers [31]. 

Advancing age increases the rate of cartilage wear. As cartilage ages, several alterations are observed, including a reduction in the chondrocyte density, a decrease in the proteoglycans aggregate size, and the overall thinning of the cartilage [9,32,33,34,35]. In addition, with age, the pore area at the surface of the bone, such as the femur, is known to increase [36], decreasing the overall fracture strength and increasing the susceptibility for the cartilage to wear. Other causes of articular cartilage damage include infections and diseases such as septic arthritis [37,38] and myopathy [39]. 

## 3. Current Strategies for Cartilage Defect Repair

Articular cartilage defects do not instantly heal, and the current treatment available for damaged articular cartilage is limited. It is important that whichever treatment is used, the joint must maintain the freedom of movement that would be expected [40]. When the cartilage damage or arthritis is mild, physiotherapy can be used to strengthen the surrounding muscles, in conjunction with anti-inflammatory drugs to reduce pain and swelling [39,41,42]. Regular exercise has also been observed to control mild symptoms [43]. However, if cartilage damage is severe enough that these techniques become ineffective at relieving symptoms, interventions such as surgery may be required.

Although not a long-term solution, pain from small defects in cartilage and early arthritis can be relieved with the use of arthroscopic lavage/debridement [44]. During arthroscopic lavage the cartilage is washed by an injection of saline solution [45], while during anthropic debridement, cartilage is smoothed and damaged fragments are removed. However, the effectiveness of these procedures is unclear [46]. 

Depending on the nature and extent of the damaged cartilage, either regeneration or replacement is possible with the use of one of several currently available techniques. One such technique is microfracture. By creating small fractures on the bone, a blood clot is formed (haematoma) containing materials from the bone marrow. The material from the bone marrow includes mesenchymal stem cells that are capable of differentiating into chondrocytes in order to create new cartilage [47,48]. This method is fast and relatively non-invasive, however, the blood clot is often unable to completely fill the defect [49]. In addition, the cartilage formed is not hyaline but rather fibrocartilage, which is denser, weaker, and has lower stiffness compared to hyaline cartilage [50]. For example, grafted hyaline cartilage stiffness at the knee was measured at 3.0 ± 1.1 N, while the fibrous tissue had only half the stiffness value (1.5 ± 0.35 N) [51], thus, resulting in an increased probability of recurring damage or deterioration at the site [52,53].

Alternatively, during osteochondral autograft transplantation (also known as mosaicplasty), cartilage and subchondral bone from a non-load-bearing region is transplanted to the load-bearing damaged site. The practical limitation of this method is the finite amount of donor site material, and so is limited to only small defects [54,55,56]. Cartilage from the low load-bearing regions may not be suitable as it has not been developed for the higher load-bearing region and more susceptible to damage. If the area of damage is too great, osteochondral allograft transplantation maybe used. This technique uses healthy cartilage, donated from a cadaver; as a result, the cartilage has the correct properties for the site. The issue with this method is that it relies on a cadaver donor, and has a possible risk of rejection from the immune system.

Autologous chondrocyte implantation (ACI) involves extracting healthy cartilage from a non-weight-bearing area for the collection and culture of chondrocytes. The cells are then introduced to the damaged site under a perichondrial flay. Alternatively, in the case of matrix-induced autologous chondrocyte implantation (MACI), a scaffold is inserted into the defect before the cells are introduced [57,58]. The advantage of this technique is the autologous nature, reducing the risk of rejection, as well as the potential of an unlimited supply of cells. However, this method is slow and requires the patient to undergo multiple surgical procedures. The scaffold can be designed to remain permanently as a support for the new matrix, however, the new cartilage would be required to form around it, deviating from its natural structure. Alternatively, the scaffold could be degradable. This would allow the cartilage to form naturally, however, current control on the polymer degradation remains insufficient. If the supporting tissue remained for too long, acting as a semi-permanent material, similar to the non-degradable scaffolds, the new cartilage would be required to form around it. In contrast, if the scaffold degraded too rapidly before cartilage could form, then the scaffold would become redundant. More details on cartilage repair methods can be found in a recent review by Hunziker *et al.* [59].

The last surgical option is a total joint replacement or resurfacing. This is used only when articular cartilage is damaged beyond repair and a patient’s symptoms warrant an irreversible surgical intervention. The survivorship of total knee joint replacement is currently between 90% and 95% at 10-years, after which failure rates increase [60,61]. Younger patients may, therefore, require multiple surgeries during their lifetime, resulting in an increase in healthcare-associated costs and surgical burden to the patient. Total joint replacements can also have other complications such as: release of debris, joint loosening, noise, and mechanical failure. These modes of failure may be reduced if a more stable implant with increased longevity could be achieved. 

Tissue engineering is another potential method for cartilage repair. So far, the general approach to cartilage tissue engineering has been to place cells into a hydrogel scaffold and culture them in the presence of nutrients and growth factors, and sometimes with the assistance of mechanical stimuli. However, such a tissue-engineered cartilage often does not have the necessary mechanical properties that have immediate load-bearing capability [62]. Although this approach has been extensively studied, it will not be covered in this article; for more information on this approach a number of reviews are available [63,64,65,66,67]. In this review, focus will be on hydrogels that have a good mechanical stiffness match and comparable mechanical performance (strength and toughness) to those of native cartilage for direct replacement and repair of damaged or diseased cartilage.

## 4. Synthetic Materials Used in Joint Replacement and Cartilage Repair

It is important that the replacement material possesses biomechanical properties similar to the surrounding native cartilage if it is to be functional. If the replacement material deforms excessively, it may not assist the surrounding cartilage with supporting applied loads. In contrast, if the material is too stiff it will bear a larger proportion of the load, increasing the probability of wear and degradation. 

The materials currently used for joint replacements are engineered materials such as: cobalt chrome (CoCr) alloys, ceramics, and ultra-high molecular weight polyethylene (UHMW-PE). These materials possess significantly different mechanical properties compared to cortical bone and articular cartilage (Table 1). Although these materials are biocompatible and non-degradable, they can induce a stress-shielding effect with the surrounding bone, except for polyethylene. The stiffness of the implant materials leads to “shielding” of the bone from the load that would normally be applied. This has an adverse effect on the remodelling of the native bone, including a reduction in its density [68], which may lead to early loosening or failure of the implant. 

Bearing surfaces exhibit wear that leads to the release of debris, which can result in: third body wear, as well as local and systemic effects. Particular concerns arise from metal-on-metal (MoM) bearings (particularly cobalt-chrome on cobalt-chrome bearings). As the first regularly used metal-on-metal bearings [71], cobalt-chrome as a hip replacement demonstrated excellent corrosion resistance, chemical inertness, and biocompatibility [72]. Similar to other metal-on-metal implants, metal ions have been reported to liberate themselves from the surface during wear [73,74,75]. *In vitro* experiments have shown cobalt ions to have genotoxic repercussions [76], and to be capable of exhibiting carcinogenic behaviour, depending on the oxidation state [77]. In addition, cobalt-chrome ions are capable of damaging DNA across cellular barriers [78], resulting in a greater potential area of damage. The released metal ions to the bloodstream and urinary tract have shown no significant link with cancer diagnosis [79,80,81,82]. Furthermore, since the development of metal-on-metal hip implant materials, long-term survival rates (10 years) have been observed [83]. However, because of the potential risks from released metal ions, the UK’s Medicines and Healthcare Products Regulatory Agency (MHRA) issued a medical device alert, with advice on the management and monitoring of patients with MoM hip implants [84]. A number of MoM implants have also been recalled from the market due to health concerns [85,86,87]. 

More commonly used contemporary implants, are metal acetabular shells with a modular or integrated polyethylene (PE) liner, which acts as the bearing surface [88]. No elevation in metal ion concentration has been observed after hip arthroplasty, even after two years post-surgery [89]. However, the PE is still susceptible to eventual wear. The materials mentioned above are used in total joint replacement and are generally used for late-stage osteoarthritis (OA) or severe cartilage damage (such as sports and accidents). 

For early-stage arthritis and small local damage to the cartilage, ACI and MACI methods are typically used. The MACI method involves the development of degradable cartilage mimicking scaffolds, which are impregnated with chondrocytes [64]. For example, using *in vitro* methods, cells that were implanted into low melting point agarose matrixes, produced large collagen concentrations within 20 days [90]. As stated previously, the primary issue with the MACI model is that the current knowledge on these materials requires further investigation to accurately understand the rate of degradation of the degradable scaffolds. Limited clinical research is available on chondrocyte impregnated implantation and the amount of additional performance and stability these materials could offer [91]. Therefore, further development is required before ACI and MACI materials can be implemented. 

Alternatively, an implant design that consists of a non-degradable cartilage mimicking polymer to permanently replace the damaged cartilage may be advantageous. Consisting of either a single polymer layer or a layered structure, surface-grafted poly(2-methacryloyloxyethyl phophorylcholine) (PMPC) on UHMW-PE, was found to supress the rate of wear, when compared to bare UHMW-PE, during stimulated conditions [92,93,94,95]. PMPC is a polymer that is both biocompatible and hydrophilic, that can also suppress biological interactions [96,97], and has been used as a surface coating on other biocompatible devices [98,99,100]. Other implant surfaces that have been investigated include: 3-dimethyl (3-(N-methacrylamido)propyl), ammonium propane sulfonate (MPDSAH) [101], and polyacrylic acid (PAA). PAA was found to be capable of reducing the PE surface friction, although it did also gradually shear off from the surface [102]. Alternatively, rather than a solid polymer block, the lubricating ability of polymer brushes has also been investigated [103]. The presence of PMPC brushes on a PE surface has been shown to reduce wear and haemolysis rates of the surrounding cells [104]. As investigations into the capability of polymers as cartilage substitutes continue, the remaining fact is that many of their properties remain estranged to articular cartilage.

## 5. Alternative Materials—Hydrogels

Hydrogels are highly hydrated polymers that have been proposed as a potential replacement material when cartilage is sufficiently damaged (from stage 3, moderate osteoarthritis), reducing its function and warranting its removal. The advantages of these materials over others described in the previous section includes their biocompatibility, exceptional lubrication ability, and low protein adsorption rates. The excellent lubrication ability is attributed to unique, multimodal lubrication mechanisms, consisting of fluid pressurisation-mediated lubrication and boundary lubrication [105,106]. This is additionally supported by an absorbed film consisting of surface-bound macromolecules, such as phospholipids, glycoproteins, proteins, and related complexes, including hyaluronic acid. In recent years, *in vivo* experiments have investigated the use of hydrogels as a treatment for damaged cartilage [107]. The issue with these materials is the lack of fracture strength and elastic modulus that is required to support the expected load. There are several methods that have been developed to strengthen and toughen hydrogels, including doping with nano- or microparticles [108,109,110,111,112,113,114,115], weaved structured meshes [116], soft filling [117], polymer structure alignment [118], and polymer entanglement [119].

**Figure 2:**
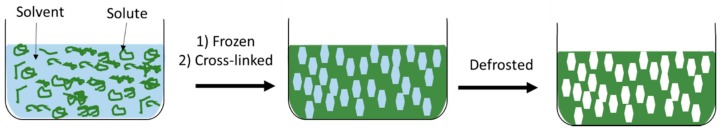
Cryogel synthesis: the polymer solute solution is frozen, encouraging a phase separation with the solvent. It is then cross-linked with an initiator to form the polymer, and then defrosted to expose the porous structure.

Cryogels are hydrogels that are synthesised by freezing the solution to encourage phase separation between the solute and the solvent (Figure 2). After freezing, the solute is cross-linked to form the polymer, while the solvent remains inert, acting as a pore-forming substance. Once de-frosted, the highly interconnected porous structure can be observed, which is later hydrated with water [120]. The structure and physical properties of the cryo-polymers can be controlled by both external and compositional conditions, including overall synthesis time [120], cooling rate [118], solute concentration [121], and degree of polymer crosslinking [122]. Polymers that have been strengthened using these methods include: poly(vinyl acid) (PVA) [123], polyacrylamide (PAAm) [120], isopropylacrylamide [118], Poly(acrylic acid) (PAA) [122], poly(ethylene glycol) (PEG) [124], agarose/alginate copolymer [125], and chitosan/gelatin [121]. These cryogels have been so successful at mimicking cartilage that they are currently being used as cartilage replacement implants. The Cartiva SCI implant is a PVA cryogel that has been used as a cartilage implant material since 2002 (excluding the USA) [126,127], and has proven to reduce pain and improve knee function [128].

Polyampholytes [129] and polyion complexes [130], also, have high fracture strength and moduli due to a high density of ionic bonding. The large number of bonds work cumulatively to generate a large fracture strength, while the ionic bonds ensure that damage can be rapidly self-healed when in water. However, more studies are required to determine how strong these ionic hydrogels would be in a biological environment, as the ionic bonding could be challenged when in the presence of many more ions [130].

**Figure 3:**
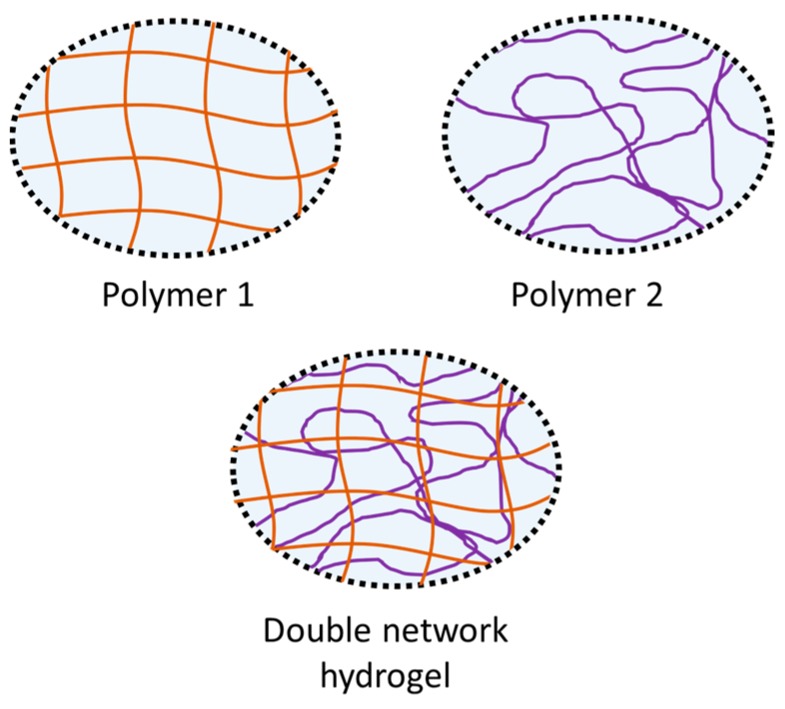
Double network hydrogels consist of a rigid polymer with a high crosslinking density for high strength. This polymer is independantly polymerised from the second polymer that is interpenetrated and crosslinked at a lower density for flexability.

Other polymers, known as double network (DN) hydrogels, use two different polymer networks, which work cooperativly to enhance the properties of the hydrogel. Once swollen in water, DN hydrogels can have typical water contents of 60–90 wt %. DN hydrogels consist of two interlocking crosslinked polymers formed by independent polymerisation reactions (Figure 3). The individual mechanical properties of the polymers are often different. The first polymer network has a higher molar ratio, and is crosslinked at a higher density in order to increase the mechanical strength of the hydrogel. In contrast, the second network is crosslinked at a lower density, and ensures the flexibility of the hydrogel [131]. By varying the properties of the individual polymer networks, the overall mechanical properties of the hydrogel, such as the elastic modulus, fracture energy, and hysteresis, can be tuned, thus, making them an attractive option as a potential articular cartilage replacement material [132,133,134,135]. Several design strategies have been identified to enable the dissipation of mechanical energy while maintaining the gel elasticity. For more details on the DN hydrogel preparation strategies, we refer the reader to a number of comprehensive reviews available on this topic [136,137,138].

A major advantage of the DN hydrogels, is the ability to finely tune their properties by alterations in the polymer molar ratio, crosslinking density, and polymer charge, since the two polymer networks are polymerised independently. Gong *et al.* found that varying the molar ratio between the 1st and 2nd network of a poly-2-acrylamido-2-methylpropanesulfonic acid (PAMPS)/PAAm DN hydrogel, the fracture stress would vary by over an order of magnitude [139], achieving an optimal fracture stress value at a molar ratio of 2 between the PAAm and PAMPS. This polymer ratio dependance on properties has also been observed in other DN hydrogels. The fracture strength of PEG/PAA DN has been reported to vary with PAA mass fractions between 0.1 and 0.9. The optimal fracture stress was reported to occur at a mass fraction of 0.4, while, in contrast, the modulus remained insensitive to the variation of the hydrogel composition [140]. In contrast, for poly(N-(carboxymethyl)-N,N-dimethyl-2-(methacryloyloxy) ethanaminium) (PCDME) and poly(2-acrylamido-2-methylpropanesulfonic) (PAMPS) DN hydrogels, increasing PCDME to PAMPS composition ratios from 10 to 38 (mol/mol), increased both the fracture stress and the Young’s modulus [141]. Furthermore, PAAm/sodium alginate DN hydrogels exhibited a wide range of fracture stress and elastic modulus values that decreased with increased PAAm concentration [142]. Similarly, Li *et al.* found that, at alginate concentrations between 2 wt % and 7 wt %, the DN hydrogel elastic modulus and fracture energy increased with increasing alginate content [134].

Varying the concentration of the crosslinkers during polymerisation is an additional method used to tailor DN hydrogel properties. By increasing the crosslinking density of the PEG polymer in a PEG/poly(methyl methacrylate) (PMMA) DN, the modulus of the overall DN hydrogel increased [143]. For an alginate/poly hyaluronic acid DN hydrogel, the compressive strength improved as the concentration of the hyaluronic acid cross linker, 1-ethyl-(3-3-dimethylaminopropyl) carbodamide (EDC), increased from 0.1 wt% to 0.3 wt% [144]. While increasing the concentration of *N*-*N’*-methylenebis(acrylamide) (MBAA), a cross linker molecule for PCDME polymerisation, Yin *et al.* found that both the Young’s modulus and fracture stress of the DN hydrogel increased [141]. The same MBAA crosslinker can be used with other polymers, such as carboxybetaine acrylamide. An increase in the MBAA concentration from 0.5 to 125 mM during polymerisation increased both the tensile strength (from 75 to 250 MPa) and modulus (from 50 to 200 kPa) [145]. 

Another method to control the DN hydrogel properties involves varying the initiator concentration used for polymerisation. In the case of PAMPS/PAAm DN hydrogels, a decrease in the initiator concentration (2-oxoglutaric acid) resulted in an increase in both the molecular weight of the PAAm and the hydrogel fracture strength [119]. Similarly, for an acrylamide/MBA DN hydrogel, increasing the redox initiator pair, ammonium persulfate and sodium metabisulfite, to 2 wt %, the overall yield of the DN hydrogel, as well as the gelation time, decreased [146].

**Figure 4:**
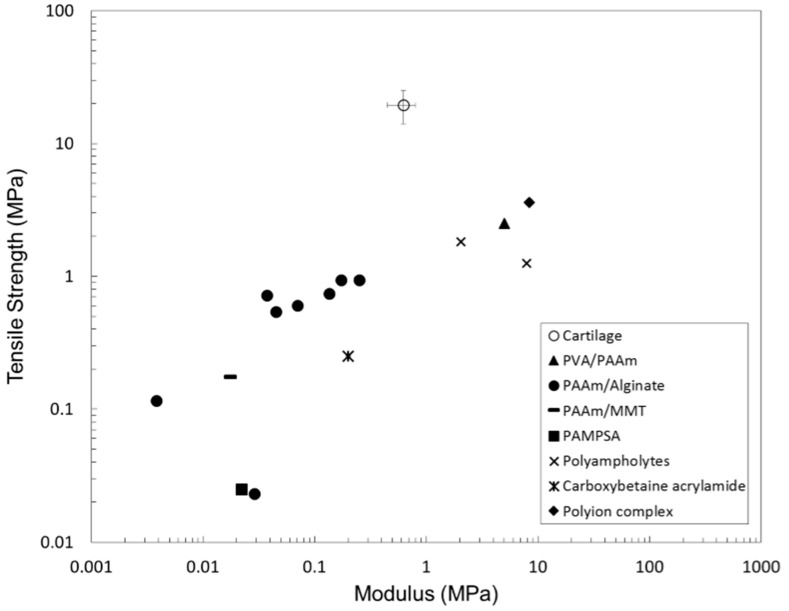
Comparison of the tensile fracture stress and Young’s modulus of damaged human cartilage [148] with those of self-healing hydrogels, including PVA/PAAm [133], PAAm/Alginate (including various ionic strengths) [142,149], PAAm/MMT [111], PAMPSA [150], Polyampholytes [129], Carboxybetaine acrylamide [145], and Polyion complexes [130].

Despite all these methods available for controlling the hydrogel properties, they remain inferior to natural articular cartilage. One such example is the difference in structure. Articular cartilage is anisotropic, resulting in different physical properties along the depth of the material, making it more adaptable under a wide range of conditions. Alternatively, DN hydrogels are typically homogenous, lacking the variation in structure that is observed in articular cartilage. Figure 4 compares the tensile fracture stress and modulus of cartilage, with self-healing hydrogels, as recently reported in the literature. The Young’s modulus of the cartilage was determined from the aggregate modulus of knee joint cartilage from five speices (bovine, canine, human, monkey, and rabbit) [147,148]. While the tensile strength values were measured with human cartilage at ages between 10 and 80 years [148], the recently reported polymers remain short of the cartilage properties. It is clear that, with the continual development of the various techniques, as discussed above, the polymer fracture strength and modulus values are continually improving. This provides a promising prospect, that hydrogels with properties similar to articular cartilage may be reported in the near future.

## 6. Hydrogel Integration 

When the physical properties of the hydrogels are refined, they can only function as a cartilage replacement once sufficiently integrated with the implant or bone surface. Current adhesion methods include the use of liners such as PE within the implant that attach via a snap-fit or locking mechanism [151]. However, these methods are not practical for softer materials, thus, alternative adhesion methods are required. Hydrogels can be designed with adhesive properties [138], although for articular cartilage applications, friction at the joint would significantly increase, resulting in stiffening at the joint and encouraging wear. Therefore, a method for attaching friction-reducing hydrogels to the bone or implant surface is also required.

Current methods for securing soft to hard materials involve using either tissue adhesives [152,153,154], or sutures and staples [155,156,157]. These methods each have their own advantages and disadvantages, depending on the situation. For instance, suturing is a more effective method when compared to sealants during spinal surgery repair [158]. In contrast, adhesives such as fibrin glue, have a greater repair ability for nerve regeneration after transecting, when compared to suturing [159].

The different fixing methods for hydrogels to aluminium were compared by Arnold *et al.* who tore PAMPS/PDMAAm and PAMPS/PAAm DN hydrogels, fixed with either an acrylic adhesive or surgical sutures (Vicryl 4/0) [160]. The sutured PAMPS/PDMAAm had an excellent tear out strength, with a maintained pull out strength value similar to nasal cartilage. However, the sutures had also affected the surrounding healthy cartilage. The damage to cartilage by suturing has been known to reduce the chondrocyte and proteoglycan concentration, and proceed to form fissures within the suture channel walls that filled with a loose avascular mesenchymal tissue [161]. Alternatively, if the damage had not propagated to the surface of the bone, chondroitin sulphate (CS) has been proposed as an alternative adhesive method [162]. During this method, CS is applied on the defect surface, followed by a hydroxyethyl methacrylate (HEMA) monomer solution, which is then polymerised to form the hydrogel. The CS covalently bonds with the polyHEMA hydrogel and remaining cartilage below, via vinyl groups. The adhesive strength of the CS was strong; during tensile and shear tests it was not the CS interface, but the hydrogel bulk, that was the point of fracturing. The tensile and shear strengths measured were 45 ± 2 kPa and 46 ± 1.7 kPa, significantly larger when compared to when the CS adhesive was not used ≤2.8 kPa and ≤6.0 kPa, respectively.

The securing ability of sutures, adhesives, and agarose hydrogels sealed with a fibrin glue was compared through their ability to repair human hip chondral cartilage flaps via an *ex vivo* environment by Cassar-Gheiti *et al.* [163]. The adhesives were the least efficient at securing the chondral flaps, which became loose after only 50 cycles during a simulated walking fatigue test. In comparison, when the flaps were sutured or repaired with the agarose hydrogel, the chondral flaps remained adhered beyond the 1500 cycle limit. All of the six sutured samples remained fixed after the 1500 cycles, however, two of the samples demonstrated reduced flap integrity from suture cutting, indicating a possible issue for long-term durability with this method. The agarose hydrogel was more deformable when compared to cartilage, posing a concern for unequal load distribution among the articular surface. Nevertheless, the agarose hydrogel adhesive method was observed to be a promising method in terms of stability for articular cartilage defect repair. 

**Figure 5:**
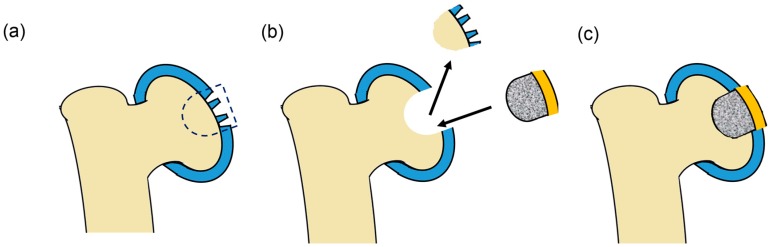
Partially interpenetrated porous implant as a replacement for the damaged cartilage area to ensure the hydrogel is secured while encouraging bone growth within the implant. This would entail (**a**,**b**) the removal of the damaged cartilage; (**b**,**c**) and the insertion of the replacement implant with the the infused replacement cartilage.

The infiltration and *in situ* polymeraisation of polymers into a porous implant, such as plugs, has also been proposed as a soft polymer fixing method (Figure 5). Using a titanium fibre mesh, Oka *et al.* partially infiltrated it with a vinyl acid solution, and polymerised, to ensured the PVA hydrogel was fully integrated [164,165,166]. However, histological changes in the opposing articular cartilage were observed, and the attachment between the PVA and the surrounding cartilage remained to be perfected [167]. The advantage of this implant design was the partial infiltration of the polymer into the implant plug, leaving the lower half porous to encourage bone ingrowth within the titanium scaffold. Once grown, this would ensure a secure fit , thus securely attaching the implant to the joint [165,166].

## 7. Summary

The development of tough hydrogels has enabled their potential as possible articular cartilage replacement materials. Hyaline cartilage is a highly complex anisotropic structure that can change its properties with time. While synthetic polymers will be unlikely to completely mimic the physical properties and stability of cartilage, their properties continue to improve. Hydrogels have been a particularly attractive perspective due to precise tunability available by their synthesis, and their intrinsic lubricating capability. 

As a permanent cartilage replacement, a hydrogel should ideally possess physical properties, such as a Young’s modulus, similar to that of the surrounding cartilage to encourage natural load distribution at the joint. If the hydrogel modulus is too high, it will not sufficiently deform and the majority of the load would be pin-pointed on the hydrogel. This would result in larger pressures, increasing the probability of the hydrogel fracturing and weakening the surrounding cartilage due to its reduced activity. Alternatively, if the modulus is too low, the hydrogel would not be able to support the load, putting more strain onto the surrounding tissue and increasing the probability of further damage.

In addition, articular cartilage is anisotropic, with different physical properties along the depth of the material. This makes the material more adaptable under a wide range of conditions. Alternatively, DN hydrogels are typically homogeneous, lacking variation in structure. More effort is needed in this area if polymeric systems are to truly mimic cartilage. 

It is also very important that the properties of the hydrogels can be tailored to match the different hyaline cartilage present in the body, which supports different amounts of load. In addition, cartilage properties also vary within the same joint, with some areas experiencing higher loads than others [168], requiring the hydrogel to be specifically designed for the site.

Finally, it is not only the material properties that need consideration, but also the integration method for enabling the hydrogels to optimally perform. A need for development in this area is evident. Further studies should also address methods for permanently attaching the implant, whether it be adhesion, suturing, infusing into a porous scaffold, or an alternative method. 

## Figures and Tables

**Table 1:** Common tensile strength and modulus values of materials used in joint repair [69,70].

Material	Tensile Strength (MPa)	Young’s Modulus (GPa)
Cortical Bone	133	17.7
Articular Cartilage	27.5	10.5 × 10^−3^
Co-Cr Alloy	1085	210
Zirconia	820	220
Alumina	300	380
Polyethylene (PE)	35	0.88

## Abbreviations

The following abbreviations are used in this manuscript:

DN	double network
ACI	autologous chondrocyte implantation
CoCr	cobalt chrome
MoM	metal on metal
UHMW-PE	ultra-high molecular weight polyethylene
PE	poly(ethylene)
PMPC	poly(2-methacryloyloxyethyl phophorylcholine)
MPDSAH	3-dimethyl (3-(N-methacrylamido)propyl) ammonium propane sulfonate
PAA	poly(acrylic acid)
PVA	poly(vinyl acid)
PAAm	poly(acrylamide)
PEG	poly(ethylene glycol)
PAMPS	poly(2-acrylamido-2-methylpropanesulfonic acid)
PCDME	poly(N-(carboxymethyl)-N,N-dimethyl-2-(methacryloyloxy) ethanaminium)
PMMA	poly(methyl methacrylate)
EDC	1-ethyl-(3-3-dimethylaminopropyl) carbodamide
MBAA	*N*-*N’*-methylenebis(acrylamide)
CS	chondroitin sulphate
HEMA	hydroxyethyl methacrylate
